# 607 Fire Safety Hazards and Burn Risks in Country: Findings from a National Cluster-Randomized Household Survey

**DOI:** 10.1093/jbcr/iraf019.236

**Published:** 2025-04-01

**Authors:** Dinasha Dahanayake, Pariwesh Raj Bista, Raslina Shrestha, Aldina Mesic, Manish Yadav, Kiran Nakarmi, Shankar Rai, Barclay Stewart

**Affiliations:** UW Burn and Wound Center; Kirtipur Hospital; Kirtipur Hospital; University of Washington; Kirtipur Hospital; Kirtipur Hospital; Kirtipur Hospital; University of Washington

## Abstract

**Introduction:**

Burn injuries remain a major public health problem in low-income countries like Country. Given the evolving landscape of cooking arrangements, electrification, and housing materials, understanding fire hazards and burn injury risks across community types (e.g., slum, urban, and rural households) and geographic zones (i.e., mountains, hills, plains) is critical for developing effective intervention bundles.

**Methods:**

A geographic zone-stratified, population-proportional, cluster-randomized, household survey of urban (n=186), slum (n=188), and rural (n=241) households (HH) across Country was performed using random walk methodology. Verbal autopsies of burn injury, fire safety surveys, and Johnson cookstove assessments were performed at each household. Descriptive statistics and chi-square tests were used to explore differences in cookstove burn-related variables across community types.

**Results:**

In the past 12 months, fire incidents differed significantly across communities (p=0.046), with the highest incidence in rural HH at 11.6%, followed by slum (7.9%) and urban HH (4.8%). Burn injuries were reported by 12% of rural HH, 5.3% slum HH, and 2.7% urban HH (p< 0.05). Flame burns were most common in rural areas (83%) while scald injuries predominated in slum (70%) and urban (80%) settings. Nearly all of slum (94%) and 79% of urban households used liquid propane gas (LPG) stoves, while 49% of rural households relied on mud stoves (p< 0.001); although 25% of rural HH also used LPG. Water was the most common fire extinguishing method (even for oil/grease fires), though 53% of rural HH reported using no tools or simply escaping (p< 0.05). Rural residents reported the most discomfort with extinguishing fires (26%), while slum residents felt the most comfortable (34.4%) (p< 0.05).

**Conclusions:**

Rural households in Country are more susceptible to burn injuries, with cooking methods and fire extinguishing strategies contributing to this elevated risk. The stark differences in distribution of cookstove type, gas leaks, and fire extinguishing methods, highlight the urgent need for targeted fire safety interventions.

**Applicability of Research to Practice:**

This research guides the creation of community-focused fire safety interventions across the country. Rural areas need better access to safer cookstoves, such as LPG stoves, and thorough fire safety education. Urban and slum areas would benefit from ongoing burn prevention education, policies for safer practices, and efforts to address hesitancy toward new regulations in slum communities.

**Funding for the Study:**

NIH Fulbright-Fogarty Fund

